# Neonatal Seizure as a Manifestation of Unrecognized Maternal Hyperparathyroidism

**DOI:** 10.4274/Jcrpe.1037

**Published:** 2013-09-18

**Authors:** Hüseyin Anıl Korkmaz, Behzat Özkan, Demet Terek, Ceyhun Dizdarer, Sertaç Arslanoğlu

**Affiliations:** 1 Dr. Behçet Uz Children Disease and Surgery Training and Research Hospital, Department of Pediatric Endocrinology, İzmir, Turkey; 2 Dr. Behçet Uz Children Disease and Surgery Training and Research Hospital, Neonatal Intensive Care Unit, İzmir, Turkey

**Keywords:** hypocalcemia, transient hypoparathyroidism, maternal hyperparathyroidism, maternal hypercalcemia

## Abstract

Maternal hypercalcemia suppresses parathyroid activity in the fetus resulting in impaired parathyroid responsiveness to hypocalcemia after birth. Resultant hypocalcemia may be severe and prolonged and rarely may lead to convulsions. Here, we present a newborn infant admitted to the pediatric emergency department at age two weeks with recurrent tonic convulsions due to asymptomatic maternal hyperparathyroidism and vitamin D deficiency. Physicians should be aware that undiagnosed maternal hyperparathyroidism can cause severe hypocalcemia in the newborn.

**Conflict of interest:**None declared.

## INTRODUCTION

During the last trimester of pregnancy, calcium (Ca) is actively transferred from mother to fetus as demonstrated by the significantly high levels of total Ca in cord blood compared to maternal serum (1). On the other hand, parathyroid hormone (PTH) and calcitonin cannot be transferred to the fetus during the last trimester. After birth, the infant’s PTH secretion, dietary Ca intake, renal Ca reabsorption, skeletal Ca stores, and vitamin D status all affect his/her serum Ca (SCa) levels. Hence, after delivery, Ca levels start to decrease and reach a nadir of 7.5-8.5 mg/dL in healthy term babies by the second day of life. This drop in postnatal SCa has been associated with hypoparathyroidism, end-organ unresponsiveness to parathyroid hormone, abnormalities of vitamin D metabolism and to hyperphosphatemia, hypomagnesemia, and hypercalcitoninemia which evolve by 12-24 hours of age (2). Maternal hypercalcemia due to hyperparathyroidism causes increased transfer of Ca to the fetus. This leads to increased fetal Ca concentrations, suppressing fetal PTH synthesis and stimulating calcitonin secretion (1,3,4). All these mechanisms result in neonatal hypocalcemia.

Here, we aimed to present a newborn with recurrent tonic convulsions as a first manifestation of transient hypoparathyroidism due to asymptomatic maternal hyperparathyroidism and vitamin D deficiency. 

## CASE REPORT

A fifteen-day-old male infant was admitted to our pediatric emergency department because of recurrent generalized tonic convulsions and apneic events associated with bradycardia and cyanosis. He was born at full term to a 24-year-old G2, P2 woman by caesarean section, following an uncomplicated pregnancy. Birth weight and length were 4050 g and 52 cm, respectively. The baby was mixed-fed (with breast milk and an infant formula). His weight at admission was 4025 g (50th-75th percentile), his height was 53 cm (50th -75th percentile), and his head circumference was 37.3 cm (50th-75th percentile). Physical examination revealed an active, afebrile baby with normal physical findings and facial appearance. Initial plasma glucose level was 91 mg/dL. Complete blood count (CBC) revealed a hemoglobin level of 14 g/dL, hematocrit 45%, white blood cell 18500/mm3 and a platelet count of 300000/mm3. SCa level was 1.5 µmol/L (normal range, 1.9 to 2.8), free Ca level was 0.64 µmol/L (normal range, 1 to 1.18), serum phosphate (P) 2.38 mmol/L (normal range, 1.2 to 2.2), alkaline phosphatase (ALP) 270 U/L (normal range, 48 to 406), and magnesium 0.8 mmol/L (normal range, 0.7 to 1.2). PTH and 25-hydroxy vitamin D [25(OH)D] levels were relatively low: 21.63 pg/mL (normal range, 15 to 65) and 6.45 ng/mL (normal range, 8 to 21), respectively. A combination of hypoparathyroidism and vitamin D deficiency were considered as the cause of hypocalcemic seizures. The baby’s Ca level reached 1.75 µmol/L with IV Ca gluconate, and he was started on 1α-hydroxyvitamin D3 in a dose of 0.25 μg/day, vitamin D3 2000 IU/day, and oral Ca lactate (50 mg/kg/day). Treatment with 1α-hydroxyvitamin D3 and oral Ca lactate were stopped at 2 months of age. The patient’s SCa, phosphate, ALP, and 25(OH)D levels returned to normal at age 3 months and the 2000 IU/day of vitamin D3 treatment was also discontinued. At age 6 months, the patient’s growth and development status as well as his Ca and 25(OH)D levels were all normal. His echocardiography performed at this time showed no cardiovascular defects.

The patient’s mother had no complaints, nor a history of major illness. Her physical examination was normal. Biochemical evaluation revealed a serum total Ca level of 2.65 µmol/L (normal range, 2.1 to 2.57). Serum P level was 1.32 mmol/L (normal range, 0.87 to 1.45), and ALP level was 155 U/L (normal range, 30 to 120). Urinary Ca was also elevated to 310 mg/day (normal range, 50-200). Renal sonography revealed nephrolithiasis at the lower poles of both kidneys. The PTH level was found to be 201 pg/mL (normal range, 15 to 65), while 25(OH)D level was low (8.05 ng/mL; normal range, 10 to 55). Parathyroid SPECT was performed with 99m Technetium-MIBI and two adenomas were demonstrated. Primary hyperparathyroidism along with vitamin D deficiency was diagnosed and surgery was planned for the adenomas.

## DISCUSSION

After a first unexplained nonfebrile seizure, laboratory testing should be considered, particularly in neonates with seizures, such as in our patient. The workup should cover electrolyte levels, including sodium, potassium, Ca, magnesium, and phosphorus. Once hypocalcemia is found, major causes of late-onset neonatal hypocalcemia should be investigated by obtaining a basic metabolic panel, liver function tests, PTH, 25(OH)D (5). A workup for hypoparathyroidism and vitamin D deficiency should be undertaken if suggested by history or initial laboratory results; such was the case in our patient. The diagnosis of hypoparathyroidism due to maternal hyperparathyroidism was made in our patient upon evaluation of maternal PTH and Ca levels. DiGeorge syndrome was unlikely in the context of normal facial appearance and absence of cardiovascular defects. In neonatal hypocalcemia, therapy should be initiated as soon as possible to prevent complications of hypocalcemia such as neuromuscular irritability, myoclonic jerks, jitteriness, seizures, and cardiac involvement such as tachycardia, heart failure, prolonged QT interval, decreased contractibility (5).

Maternal hypercalcemia due to unrecognized hyperparathyroidism suppresses parathyroid activity in the fetus, resulting in impaired parathyroid responsiveness to hypocalcemia after birth (1,2,3,4). Hypocalcemia may be severe and prolonged. Our patient presented with late-onset neonatal seizure due to hypocalcemia secondary to hypoparathyroidism on the 15th postnatal day. This unusually late and interesting presentation had rarely been reported. Two siblings with transient hypoparathyroidism presenting with hypocalcemic seizures during the first 2 weeks of life were reported previously. In these patients, subsequent investigation had revealed an unrecognized normocalcemic hyperparathyroidism with nephrocalcinosis in the mother; the maternal hyperparathyroidism was found to be caused by two parathyroid adenomas (6). Most infants with neonatal hypocalcemia due to maternal hyperparathyroidism require short-term Ca supplementation for 3 to 5 months, at which time the hypoparathyroidism resolves (3,4). Ca supplementation was required for 2 months in our patient.

Maternal Ca, phosphorus and PTH levels were assessed to help determine the cause of hypocalcemia in our patient. The diagnosis of maternal primary hyperparathyroidism along with vitamin D deficiency was established. In maternal primary hyperparathyroidism, it is always difficult to make an early diagnosis during pregnancy as the symptoms are usually subtle and are easily confused with other minor complications of pregnancy. The majority of patients with primary hyperparathyroidism have mild hypercalcemia, while about 5% of them are normocalcemic. Thus, it is necessary to assess maternal PTH levels in addition to Ca and phosphorus levels during investigation of neonatal hypocalcemia (6,7). Hyperparathyroidism in asymptomatic mothers might easily have been missed if the maternal Ca status had not been investigated (3,4). Such was the case in our patient. Maternal Ca and phosphorus levels should be routinely evaluated during pregnancy.

However, maternal hyperparathyroidism may lead to specific complications including nephrolithiasis, pancreatitis, gastrointestinal ulcers, and life-threatening hypercalcemic crisis (8,9). Our patient’s mother had primary hyperparathyroidism with hypercalciuria and nephrocalcinosis. These abnormalities may also affect the fetus and may lead to various complications including intrauterine growth restriction, low birth weight, preterm delivery, intrauterine death and neonatal hypocalcemic tetany and seizures (10).

The findings in our patient indicates, once again, that undiagnosed maternal hyperparathyroidism causes severe hypocalcemia in the newborn and that physicians should be aware of this pathology. When newborns present with hypocalcemic seizures, appropriate investigations should be carried out to exclude maternal hyperparathyroidism.
